# Bankruptcy prediction using ensemble of autoencoders optimized by genetic algorithm

**DOI:** 10.7717/peerj-cs.1257

**Published:** 2023-06-08

**Authors:** Róbert Kanász, Peter Gnip, Martin Zoričák, Peter Drotár

**Affiliations:** 1 Department of Computers and Informatics, Faculty of Electrical Engineering and Informatics, Technical University of Košice, Košice, Slovakia; 2 Department of Finance, Faculty of Economics, Technical University of Košice, Košice, Slovakia

**Keywords:** Autoencoder, Bankruptcy prediction, Imbalanced learning, Neural networks, Genetic algorithm, Financial ratios

## Abstract

The prediction of imminent bankruptcy for a company is important to banks, government agencies, business owners, and different business stakeholders. Bankruptcy is influenced by many global and local aspects, so it can hardly be anticipated without deeper analysis and economic modeling knowledge. To make this problem even more challenging, the available bankruptcy datasets are usually imbalanced since even in times of financial crisis, bankrupt companies constitute only a fraction of all operating businesses. In this article, we propose a novel bankruptcy prediction approach based on a shallow autoencoder ensemble that is optimized by a genetic algorithm. The goal of the autoencoders is to learn the distribution of the majority class: going concern businesses. Then, the bankrupt companies are represented by higher autoencoder reconstruction errors. The choice of the optimal threshold value for the reconstruction error, which is used to differentiate between bankrupt and nonbankrupt companies, is crucial and determines the final classification decision. In our approach, the threshold for each autoencoder is determined by a genetic algorithm. We evaluate the proposed method on four different datasets containing small and medium-sized enterprises. The results show that the autoencoder ensemble is able to identify bankrupt companies with geometric mean scores ranging from 71% to 93.7%, (depending on the industry and evaluation year).

## Introduction

The complexity of economies has increased in recent decades, and companies are relying more on their business partners. A failure of a particular company may have a considerable effect on its business partners, and as such, it is frequently important to have information about the potential for bankruptcy. The topic of bankruptcy prediction has been studied for a few decades. The goal is to predict whether a company will go bankrupt or not. Bankruptcy prediction may take the form of binary outcomes, ratings, or scores. The definition of bankruptcy is crucial for identifying a firm as bankrupt. It is usually defined by bankruptcy laws, which provide a legal framework and may differ among countries. We should distinguish between the following terms: liquidity, insolvency, and bankruptcy. The relationships between them were analyzed in Bryan, Tiras & Wheatley (2002). These terms may be used with different meanings depending on the context, but the following definitions are the most common. Liquidity represents a firm’s ability to convert its assets to cash (or its equivalent) to fulfill short-term liabilities to creditors. Insolvency means that a firm is not able to repay its debt when it is due. If a firm is not able to resolve liquidity or solvency issues, it may lead to filing for bankruptcy, which is a legal act indicating serious financial problems. Both the lack of liquidity and insolvency may indicate the potential for bankruptcy in advance.

The most common source of information about firms is financial reports, which offer structured information regarding assets, liabilities, costs, revenues, and eventual profits or losses. They are published either quarterly or yearly. A company can exhibit some initial indicators of upcoming bankruptcy several years prior to the bankruptcy filing. However, in some industries, even data from the current year do not possess any indications of potential problems. Thus, several studies have analyzed the optimal number of time periods prior to bankruptcy for obtaining the most accurate bankruptcy predictions (*e.g*., Tian & Yu, 2017; Volkov, Benoit & Van den Poel, 2017; Ben Jabeur, 2017; Alminos, del Castillo & Fernandéz, 2016). Financial ratios, which are based on financial reports, are frequently used due to their better comparability. In general, financial ratios may be divided into four main categories: liquidity, profitability, activity, and solvency (*i.e*., Cultrera & Brédart, 2016; Liang et al., 2016). For small or medium-sized companies, there might be a government-run register for annual reports or commercial databases. Some models (Ciampi et al., 2021) also include macroeconomic data such as GDP, inflation, or market information (Li & Faff, 2019).

Over the past few decades, many authors have approached the issue of bankruptcy prediction from many different viewpoints by employing various methods. Arguably, the most popular method is Altman’s Z score (Altman, 1968), which has been subsequently reevaluated (Altman et al., 2017). Altman’s model was one of the first to employ statistical methods for bankruptcy prediction. Development in the fields of artificial intelligence and machine learning has also inspired the application of these novel approaches for bankruptcy prediction. Jacobson & Von Schedvin (2015) analyzed how corporate failures can be propagated through trade credits from customers to suppliers. Another study by Berloco et al. (2021) addressed spillover effects across a transaction network and how losses can be propagated from one firm to another.

In this article, we introduce a new bankruptcy prediction approach for strongly imbalanced datasets. To solve the imbalanced learning task, we propose an ensemble of autoencoders optimized by a genetic algorithm (GA). The ensemble creates robustness in its classifier decisions and proves to be very effective for imbalanced data. A GA is used to optimize several base classifiers in an ensemble and to tune the threshold values of autoencoders. An evaluation of four different industrial sectors shows that this approach is able to identify bankrupt companies while correctly classifying nonbankrupt companies. The proposed framework can be embedded into a decision support system for financial analysis.

The rest of the article is organized as follows. In the next section, we present a brief review of related articles on bankruptcy prediction. “Data” offers a brief description of the employed datasets. Next, “Proposed Methodology” describes the utilized methods and presents the proposed model. “Numerical Experiments” summarizes the achieved results. Finally, the discussion and conclusions are given in the last section.

## Literature review

A plethora of bankruptcy prediction articles have been summarized in multiple review articles comparing various aspects of the problem. The main highlights from the selected review articles are presented below. Kumar & Ravi (2007) analyzed studies between 1968 and 2005. They concluded that over the years, statistical methods (such as logistic regression (LR), linear discriminant analysis (LDA), quadratic discriminant analysis (QDA), or factor analysis) have gradually been outperformed in terms of prediction performance by intelligent methods. The best results were found for neural networks (NNs), namely, backpropagation NNs (BPNNs) (Vellamcheti & Singh, 2020). Alternatively, decision trees (DT) may be used, as they offer identical performance and produce easily interpretable rules. Additionally, case-based reasoning (CBR) and a rough set (RS) were considered. However, these approaches proved to be inferior to the BPNN. Support vector machines (SVMs) offer comparable accuracy to BPNNs and have two advantages: they do not suffer from local minimum problems and can be trained on small training sets. Based on combinations of multiple intelligent methods, ensemble classifiers have been built. They outperform individual methods in the majority of cases. Moreover, the authors saw an opportunity to improve bankruptcy prediction by exploring NN architectures and by further developing soft computing architectures. Smiti & Soui (2020) proposed a novel method called BSM-SAES, which combines the borderline synthetic minority oversampling technique (SMOTE) and a stacked autoencoder NN. By using BSM-SAES, they tried to extract nonlinear patterns from financial datasets to achieve improved classification performance. Even though BSM-SAES outperforms the other methods, it is important to mention, that the proposed model achieved a worse training time performance due to a large amount of time spent in extracting the important features for the classification. Ben Jabeur, Stef & Carmona (2022) proposed a model that employs the XGB algorithm for bankruptcy prediction with feature selection in the preprocessing phase. They used the financial ratios of French companies and considered a timeframe from 1 year up to three years before bankruptcy. Based on their findings, data 1 year before bankruptcy provide the best results.

Verikas et al. (2010) provided a survey on hybrid and multiple ensemble-based soft computing techniques. They found that conducting a comparison with individual techniques was difficult due to the size and number of datasets used in the analyzed articles. The authors suggested using nonfinancial features (macroeconomic data or qualitative variables) alongside financial ratios, which should yield increased prediction accuracy. However, the use of a large number of features may decrease the predictive power of the resulting model. The authors proposed using GAs or RSs for feature selection, although using a GA may be time-consuming in some cases. Another drawback of ensemble-based techniques is transparency which is rather limited when compared to RS or IF-THEN rules-based approaches.

Sun et al. (2014) reviewed the state-of-the-art modeling and sampling techniques used in the areas of financial distress and corporate failure. The authors did not compare the results of different studies. However, they categorized articles into the following topics: definitions of financial distress, sampling, and modeling methods. They found that ensemble methods are popular, yet there are still some areas that need to be further explored (*e.g*., how candidate classifiers are evaluated and selected). They also noted the class-imbalanced nature of data for financial distress prediction. Techniques such as oversampling, undersampling, or hybrids of both may be used to overcome this issue.

Alaka et al. (2018) analyzed bankruptcy prediction models from 49 studies based on multiple discriminant analysis (MDA), CBR, DTs, LR, NNs, SVMs, RS, and GAs. They defined 13 criteria and divided them into three categories (results-related criteria, data-related criteria, and tool property-related criteria), based on which they compared individual models. According to their results, NNs and SVMs offered the best results in terms of accuracy, although each method had its strengths and weaknesses. A considerable weakness for the NNs and SVMs was their ‘black-box’ nature, where the results or underlying processes are not interpretable. From the end-user perspective, models generating decision rules (*i.e*., DTs) may be appealing due to their ease of interpretation. In addition, the authors saw a future in hybrid models, where a combination of various approaches may amplify the strengths and suppress the individual shortcomings of individual methods.

Qu et al. (2019) reviewed deep learning techniques utilized for bankruptcy prediction. They argued that methods such as convolutional NNs provide superior results and take not only numeric but also textual data into consideration. Moreover, Mai et al. (2019) proved that deep learning approaches effectively integrate the incremental information derived from textual data with numeric information and achieve better prediction accuracy than methods using a single form of input. The authors also mentioned that deep learning models are difficult to interpret. The efficiency of autoencoders in terms of bankruptcy prediction was proven by Soui et al. (2020), where stacked autoencoders with the softmax classifier noticeably outperformed reference methods such as an SVM, a DT, LR, and an NN. Again, the authors pointed out the problem of interpretability, which is crucial in the field of bankruptcy prediction (Aljawazneh et al., 2021) also compared various deep learning techniques for bankruptcy prediction tasks. The application of a multilayer perceptron with six layers combined with the SMOTHE_ENN balancing technique was determined to be the best method according to various metrics and its lowest misclassification rate.

The accuracies of models may vary depending on the analyzed country, the industry, and the sizes of companies. Prusak (2018) compared bankruptcy prediction studies in selected Central and Eastern European countries. He concluded that research in Poland, the Czech Republic, and Slovakia is comparable to high-quality research in the rest of the world. Gregova et al. (2020) compared various statistical and intelligent methods on a dataset of Slovak firms. Their results favored intelligent methods over classic statistical methods, with an NN as the most accurate method in general. Kovacova et al. (2019) analyzed variables for use in bankruptcy prediction models for Visegrad group countries. They concluded that models are usually constructed for a specific industry and are therefore sensitive to that industry. The authors listed the following financial ratios as those that are most commonly used in Slovakia: the current ratio, cash ratio, liabilities/total assets ratio, equity/total assets ratio, and return on assets.

Bankruptcy prediction data are notoriously imbalanced (*i.e*., Ghatasheh et al., 2020; Veganzones & Severin, 2018; Le et al., 2018; Zhou, 2013). Based on the data from the Statistical Office of the European Communities (2020) for selected member states of the European Union, the ratios of closures to several existing companies are between 1.45% and 11.88%. The standard approach for imbalanced datasets is to resample the data. It is possible to either oversample the minority class (in our case, bankrupt companies) or to undersample the majority class (going concern businesses). There are, however, other methods, such as the cost-sensitive method, which was used by Ghatasheh et al. (2020).

The quality of the constructed model is usually not determined only by the number of correctly identified bankrupt companies out of all companies in the set but also by the ratio of misidentified companies (nonbankrupt companies identified as bankrupting and *vice versa*). Thus, models are usually compared based on some combination of the following statistics: the type I error, type II error, sensitivity/recall, specificity, geometric mean (GM), and area under the receiver operating characteristic curve (ROC AUC) (Dastile, Celik & Potsane, 2020). According to Luque et al. (2019), the GM is one of the best metrics for imbalanced class data, although it only focuses on successes and dismissing errors.

## Data

In this study, we utilized a bankruptcy dataset (Drotár et al., 2019) composed of the financial ratios of thousands of small and medium-sized enterprises (SMEs) operating in the Slovak Republic during 2010–2016. Data were acquired from publicly accessible financial statements. Each company was characterized by financial ratios based on information acquired from annual reports three years prior to the evaluation year ( ${R_{eval}}$). For the evaluation year ${R_{eval}}$, we considered a year in which the company was evaluated as either a going concern (nonbankrupt) or financially distressed (bankrupt). The utilized bankruptcy dataset offered four ${R_{eval}}s$, namely, 2013, 2014, 2015, and 2016. The actual financial status of a particular company in a particular ${R_{eval}}$ was expressed *via* 20 financial attributes regarding the activity, liquidity, profitability, and solvency of the company, which are listed in Table 1. For analyzing the ability of the constructed model to identify bankrupt/nonbankrupt companies, we also used combinations of the available data up to three years prior to the particular ${R_{eval}}$. For example, in the case of ${R_{eval}}$ = 2013, we used data one year prior to the evaluation year ( ${R_{eval - 1}}$), *i.e*., data from 2012. Then, we combined data from one (2012) and two (2011) years prior to the evaluation year. This is denoted as ${R_{eval - 2}}$. Finally, we combined all available data (2012 + 2011 + 2010) for a particular evaluation year to create a dataset denoted as ${R_{eval - 3}}$. An overview of the data variants used for ${R_{eval}}$ = 2013 is depicted in Table 2. Taking a combination of data from two ( ${R_{eval - 2}}$) and three years ( ${R_{eval - 3}}$) prior to bankruptcy into account, we obtained 40 and 60 features, respectively.

**Table 1:** List of financial ratios based on their financial categories.

Category	Financial ratio
Activity	Total asset turnover
	Asset turnover days
	Total days with receivables outstanding
	Inventory turnover days
Profitability	Return on assets
	Return on equity
	Return on sales
	Return on investment
	Labor-to-revenue ratio
	Wages-to-added value ratio
	Labor productivity
Liquidity	Cash ratio
	Quick ratio
	Current ratio
Solvency	Debt-to-assets ratio
	Debt-to-equity ratio
	Financial leverage
	Debt-to-income ratio
	Debt service coverage ratio
	Asset coverage ratio
	Bank liabilities-to-debt ratio

**Table 2:** Overview of the data variants utilized in the experimental study for ${R_{eval}}$ = 2013.

2010	2011	2012	No. of features
		${R_{eval - 1}}$*	20
	${R_{eval - 2}}$**		40
${R_{eval - 3}}$***			60

**Note:**

The asterisks graphically indicate the number of features used in experiments. The number of asterisks is proportional to the number of features.

The data originated from four different industries, namely, agriculture, construction, manufacturing, and retail. An overview of the detailed data distribution for each evaluation year and industry is depicted in Table 3. The descriptive statistics of the financial ratios are stated in Zoričák et al. (2020). As we can see, the number of bankrupt companies is significantly smaller than the number of nonbankrupt companies. This process forms a binary classification task with a severely skewed data distribution.

**Table 3:** Distribution of bankrupt and nonbankrupt samples for the utilized industries for ${R_{eval - 1}}$.

	${R_{eval}}$			
	2013	2014	2015	2016
Agriculture	6/1,442	6/1,622	8/1,882	8/1,991
Construction	25/1,709	30/2,165	7/6,327	4/6,263
Manufacturing	30/4,077	30/4,450	26/5,019	14/5,840
Retail	11/5,195	11/6,107	7/6,327	4/6,263

**Figure 1:**
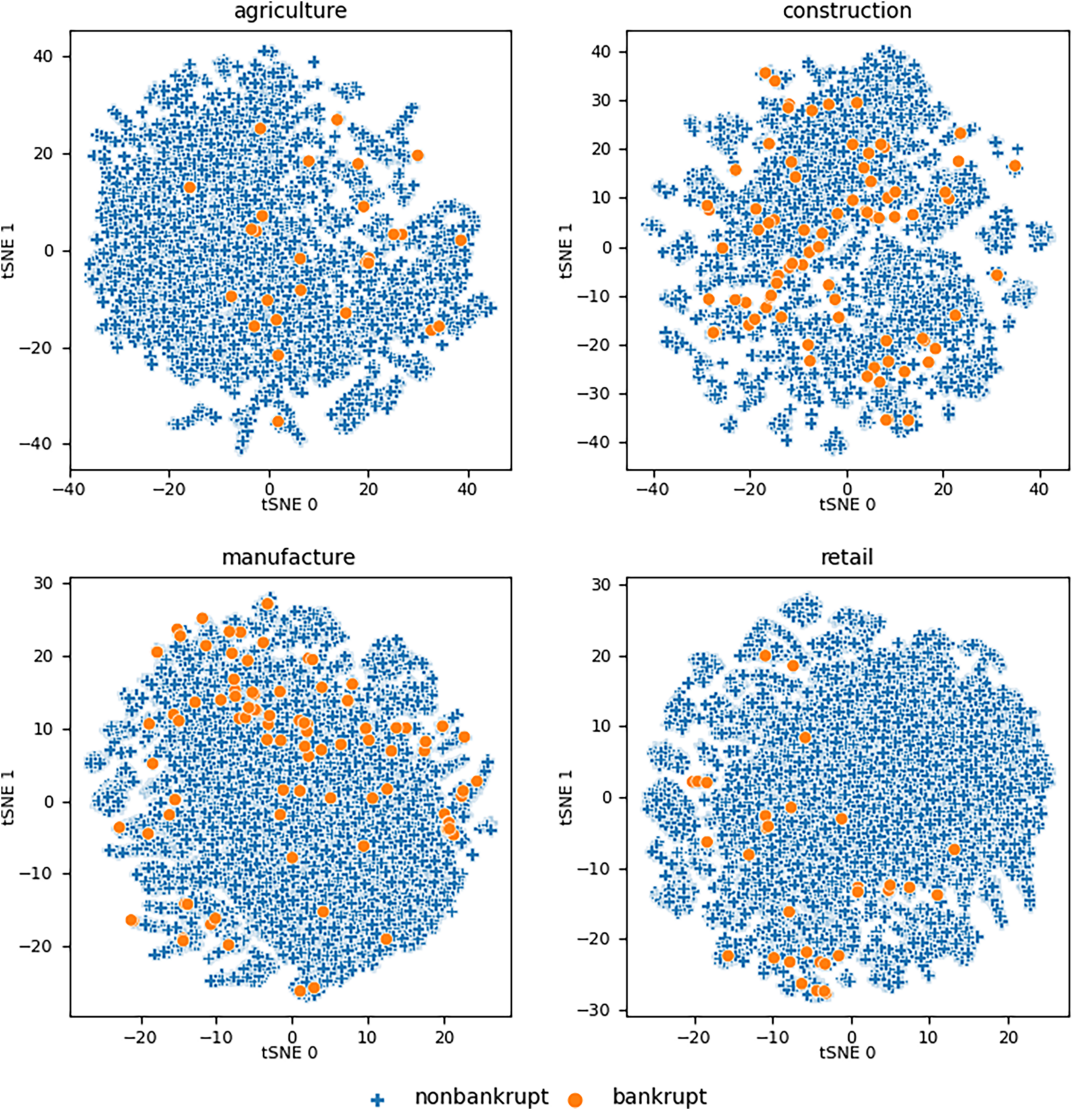
Visualizations of the datasets using t-SNE for all utilized ${R_{eval}}s$ considering features from all three years prior to ${R_{eval}}$.

The initial overview of the data distribution was obtained by using the t-distributed stochastic neighbor embedding (t-SNE) technique (Van der Maaten & Hinton, 2008). t-SNE is a statistical method for visualizing high-dimensional data by giving each data point a location in a two- or three-dimensional map. Figure 1 shows the maps of the two-dimensional representations of the utilized datasets with 60 features for a particular industry. As we can see, bankrupt companies form clusters that overlap with the clusters of nonbankrupt companies. This leads to the hypothesis that the use of a linear classifier is not suitable for solving this scenario.

## Proposed methodology

The proposed approach is based on an autoencoder ensemble with an autoencoder threshold optimized by a GA. First, we introduce a bankruptcy detection method based on a single deep autoencoder (SDA). Then, we introduce two approaches based on an ensemble of shallow autoencoders, namely, a single-threshold autoencoder ensemble (STE) and a multiple-threshold autoencoder ensemble (MTE).

### Autoencoders

An autoencoder is a type of artificial NN that is trained in an unsupervised manner by a backpropagation algorithm (Kramer, 1991). It learns how to efficiently compress data to a lower-dimensional representation (encoding) and reconstruct them back to a representation that is as close as possible to the original data representation by capturing the most important features (decoding). By design, an autoencoder performs hierarchical dimensionality reduction on the input data, similar to principal component analysis (PCA) (Chaurasia, Goyal & Rajput, 2020). The major difference between PCA and autoencoders lies in the transformation part. While PCA uses linear transformations, autoencoders are based on nonlinear transformations.

Typically, an autoencoder consists of two symmetric NNs connected by a latent layer (sometimes also called a bottleneck). The latent layer represents the compressed knowledge of the original input. It prevents the autoencoder from memorizing the input data and overfitting the data. The number of nodes in the output layer is the same as the number of nodes in the input layer. The architecture of the autoencoder is symmetric. The number of nodes per layer decreases with each subsequent layer of the encoder and increases again in the decoder.

The simplest autoencoder architecture has only one hidden layer. The encoder part (* $h$*) of the hidden layer can be defined by the following equation:

(1) $${h} = \sigma ({W_{xh}}x + {b_{xh}}),$$

where *W* and *b* represent the weight and bias of an NN, respectively, and *$\sigma$* is a selected nonlinear activation function. This equation maps the input vector (***x***) into a hidden representation (***h***) using a nonlinear transformation *via* an activation function. On the other hand, the decoder part tries to reconstruct the original input (***x***) using the same transformation as that utilized during the encoding phase. This can be expressed as

(2) $${z} = \sigma ({W_{h{\bf{x}}}}h + {b_{h{\bf{x}}}}).$$

The difference between the original input and the reconstructed output is called the reconstruction error and is defined as *$||{x} - {z}||$*. The reconstruction error is a crucial metric for identifying outliers contained in the data. The autoencoder learns the distribution of the majority of observations from the input dataset. Then, the data points that are not complying with the majority of observations have higher reconstruction errors. It is also important to note that the autoencoder is trying to minimize the reconstruction error during the learning phase.

The ability to reconstruct input data *via* the STE, MTE, and SDA was measured by the mean squared error (MSE) loss function. This function computes a risk metric corresponding to the expected value of the quadratic error. The MSE can be defined by the following equation

(3) $$MSE = {1 \over n}\sum\limits_{i = 1}^n {{{({Y_i} - \widehat {{Y_i}})}^2}} ,$$

where *Y* and $\hat Y$ represent the input and predicted values, respectively. The MSE can also be utilized as an outlier score. Here, observations with scores that exceed the specific threshold can be considered outliers. This can be expressed by the following formula:

(4) $$c\left( {MSE} \right) = \left\{ {\matrix{
   1 & {{\rm{if}}\,{\rm{MSE}} > {\rm{threshold}}}  \cr 
   0 & {{\rm{if}}\,{\rm{MSE}} \le {\rm{threshold}}.}  \cr 

 } } \right.$$

To identify samples that represented bankrupt companies, an autoencoder was trained to reconstruct nonbankrupt samples with reconstruction errors that were as small as possible. For bankrupt samples, the reconstruction error was expected to be significantly higher.

### SDA

**Figure 2:**
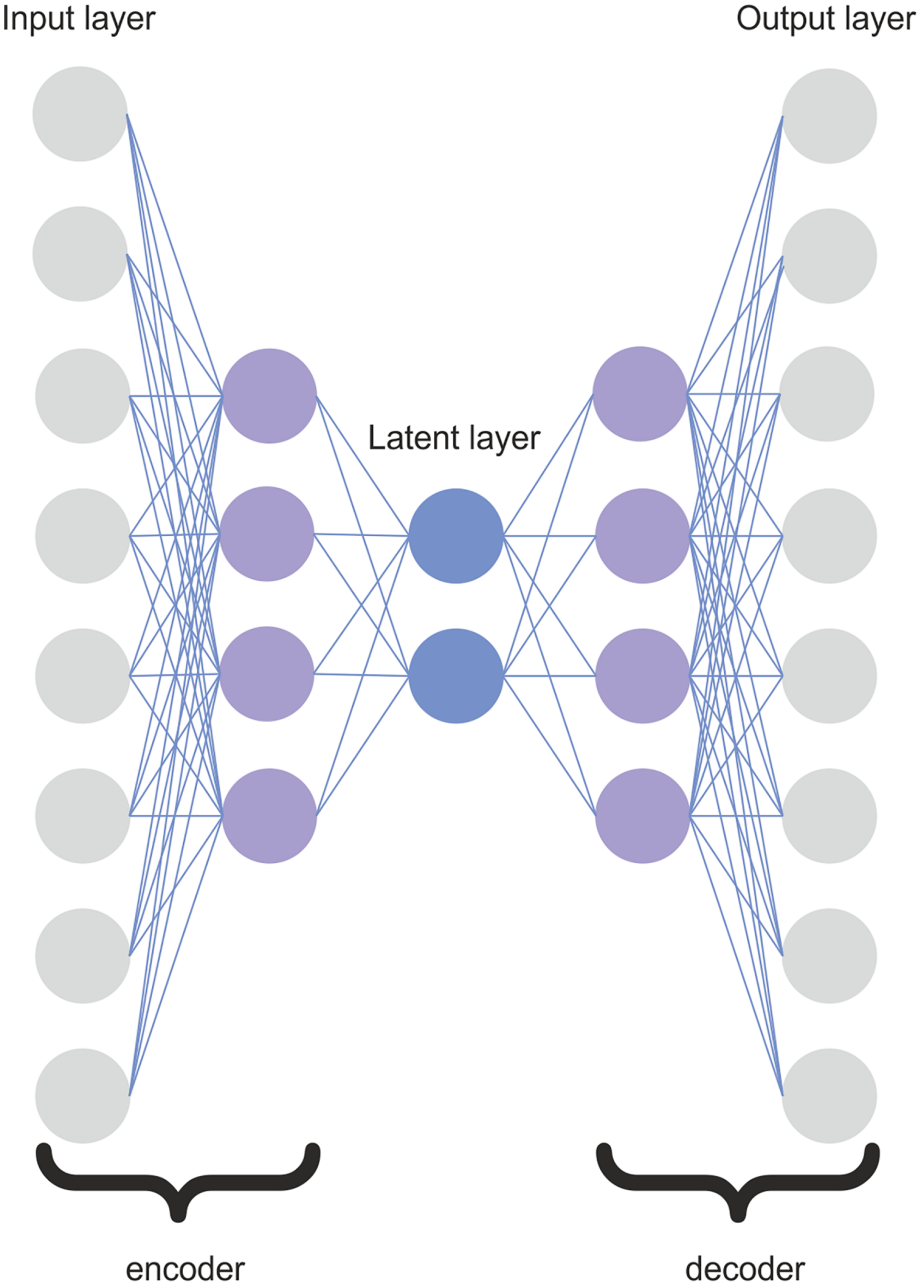
Architecture of the SDA.

The architecture of the proposed SDA is depicted in Fig. 2. The SDA consists of five layers: two layers for the encoder, two layers for the decoder, and one latent layer. The encoder is composed of one input layer and one hidden layer. The architecture of the decoder is symmetric to the encoder. The number of SDA layers was determined experimentally. Adding more hidden layers could potentially allow the autoencoder to learn more complex features. However, the use of too many hidden layers would be likely to overfit the inputs, and the autoencoder would not be able to generalize well. It would just the copy input to the output instead of learning the most representative features transformed to the lower dimension. If we denote $n$ as the number of input features, the hidden layers in the encoder and decoder have $[n/2]$ nodes, and the latent layer has $[n/4]$ nodes. We investigated several architectures, and the proposed SDA architecture yielded the best accuracy results. Therefore, for conciseness, we only describe the best architecture in this article.

The objective of the training phase was to minimize the reconstruction error. During the prediction phase, the reconstruction error of the model was compared to the decision threshold to determine whether a particular data sample belonged to a bankrupt or nonbankrupt company. The value of the threshold was determined experimentally.

To prevent overfitting, we utilized dropout regularization. Dropout is a regularization approach that prevents overfitting by ensuring that no units are codependent with one another. The main idea behind dropout regularization is that during the training process, some layer outputs are randomly ignored. We set the value of the dropout rate to 0.25. Another important part of an autoencoder architecture is the choice of the activation function for each layer. We chose the rectified linear unit (ReLU) activation function for the hidden layers and the hyperbolic tangent function for the latent and output layers.

### Ensemble of autoencoders

Ensemble learning is a technique where the outputs of multiple independent models (also known as weak learners) are combined to achieve better predictive performance than that of each individually trained model. Recent research on ensemble learning (Zimek et al., 2013) has proven its usefulness in unsupervised anomaly detection.

We proposed two different approaches for identifying bankrupt companies, namely, an STE and an MTE. In both cases, the same autoencoder architecture was utilized. The architecture was composed of the simplest shallow autoencoder with only three layers. Each weak learner processed eight randomly selected features and had four nodes on the latent layer. This set of features for a particular classifier remained constant during the experiments. The activation functions, the ReLU function for the latent layer and the hyperbolic tangent function for the output layer, were also determined experimentally.

We used three different ensembles consisting of 100, 66, and 33 shallow autoencoders. The number of weak learners depended on the set of available financial attributes in the particular dataset to ensure sufficient autoencoder diversity. For the datasets composed of 60 financial attributes, an ensemble of 100 autoencoders was applied. We also performed experiments with 40 and 20 financial attributes using 66 and 33 shallow autoencoders, respectively.

#### STE

**Figure 3:**
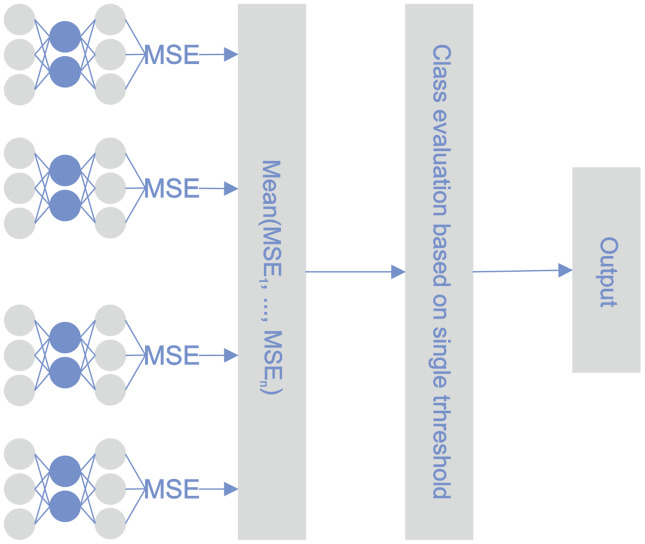
STE pipeline.

During the learning phase, the reconstruction error was minimized for every single classifier and for every observation in the training set. During the testing phase, the reconstruction error of every autoencoder was calculated for a particular observation. The determinative reconstruction error was derived by averaging the reconstruction errors of the various autoencoders. A sample with a reconstruction error above the threshold was classified as an outlier, *i.e*., a bankrupt company. The threshold value was determined experimentally. Figure 3 depicts the proposed STE architecture.

#### MTE

**Figure 4:**
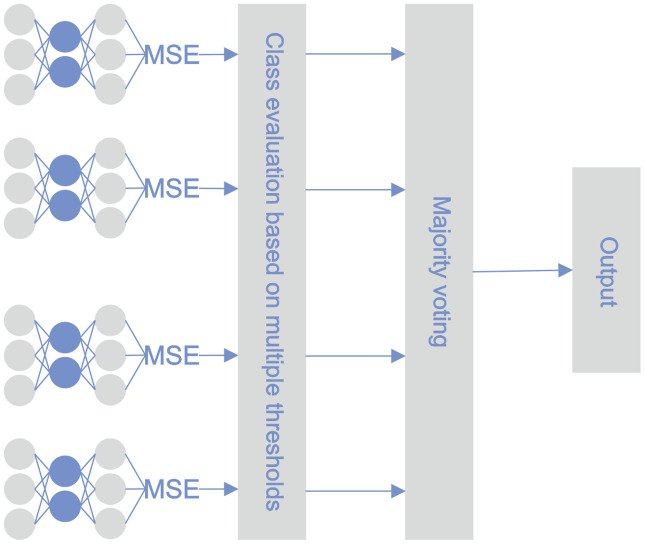
MTE pipeline.

The architecture of the proposed methodology was similar to the STE architecture; the difference lies in the approach for utilizing the base classifiers. During the testing phase, the reconstruction error was calculated for every sample using every base classifier. Bankruptcy classification was performed on every active base classifier with its threshold. After optimization, not all of the classifiers remained active. The majority vote principle determined the final classification. Figure 4 illustrates the classification pipeline after threshold optimization.

Threshold optimization was performed using a GA (Holland, 1975; Chen et al., 2020). GAs are adaptive heuristic optimization algorithms inspired by Darwin’s theory of natural selection. They use population-based search, which utilizes the concept of “survival of the fittest”. The optimization process starts by generating a random set of individuals called a population. Every single individual represents a potential solution and consists of a set of parameters known as genes. In this phase, the individuals are usually called chromosomes. The GA-based optimization process consists of seven phases (Burke et al., 2005).

1. **Population initialization**–A random population of candidate solutions is created across the search space. The order of the genes in chromosomes matters.
2. **Evaluation**–Once the population is created, a fitness score is evaluated for every single chromosome in the population. The fitness scores help to select the individuals who will be used for reproduction.
3. **Selection**–This phase involves selecting parents that mate and recombine in the next phase to create offspring for the next generation. The higher the fitness is, the higher the probability that a chromosome will be chosen for mating. The main idea of selection is to prefer better solutions over worse solutions. Many selection procedures are available, such as roulette wheel selection, stochastic universal sampling, tournament selection, rank selection, and random selection.
4. **Recombination**–This is the significant main phase in a GA. For each pair of parents to be mated, a crossover point is chosen at random from within the genes. There are three major types of crossover: single-point crossover, two-point crossover, and uniform crossover. Every pair of parents creates two new offspring, and new offspring are added to the population.
5. **Mutation**–While recombination combines parts of two parental chromosomes to create new chromosomes, mutation modifies offspring locally but randomly. This means that some of the genes in the chromosome can be modified with a low random probability. Mutation addresses population diversity and reduces the risk of premature convergence.
6. **Replacement**–As new chromosomes are formed, the chromosomes with the lowest fitness values die, providing space for new offspring because the population has a fixed size. Many replacement strategies are available, such as elitist replacement, generationwise replacement, and steady-state replacement.
7. **Termination**–The optimization process is terminated when the termination condition or multiple conditions are met. Several termination conditions can be used:

(a) There is no improvement in the solution quality after completing a certain number of generations (set beforehand).
(b) A hard and fast range of the number of generations or time is reached.
(c) An acceptable solution is obtained.

## Numerical experiments

The experiments were repeated 20 times, and the final result was an average of all 20 loops. In every iteration, the data of nonbankrupt companies were divided into training data (80%) and testing data (20%). All samples of bankrupt companies were exclusively used during the testing phase. For the baseline machine learning algorithms (the SVM and DT) and the ensemble boosting method XGBoost (XGB), 5-fold stratified cross-validation was used.

Small enterprises do not maintain their accounting records precisely, which may result in the occurrence of missing values. According to this fact, it was necessary to utilize some data cleaning operations. The missing values were replaced with the mean value of the particular financial attribute. The number of replaced values per feature was not higher than 5% for the majority of the utilized financial attributes. In some cases, the numbers of missing values were slightly higher, namely, the return on sales (11.4%), days with total receivables outstanding (11.5%), asset coverage ratio (28.19%), and inventory turnover days (30.31%). The data were standardized on a per-feature basis to have a zero mean and unit variance.

Every proposed model had some hyperparameters, such as the batch size, the number of epochs, and the numbers of layers and nodes in each layer, that needed to be set before the experiments were started. The number of epochs was set to 500, and this value remained the same for all of the experiments. The batch size was set to the number of samples in the training set for the particular experiment. The learning rates were set to 0.01.

For experiments using an ensemble with multiple thresholds, we used a GA for threshold optimization. Because of the nature of the optimization problem, we used populations with 600, 400, and 200 chromosomes and 200, 132, and 66 genes (half of the genes were for the thresholds, and half of the genes were for the active flags) in each of the chromosomes. After 400 iterations, we obtained a combination of active flags and thresholds, which we used for scoring. The active flags were used to obtain the best combination of base classifiers from the whole set. Only the base classifiers with the active flag were used in the classification process. It is important to mention that we used the GM of the test set as a fitness function.

We utilized the GM and ROC AUC as classification performance measures. From a statistical point of view, the GM is considered one of the most reliable metrics for working with skewed data distributions (Helal, Haydar & Mostafa, 2016; Luque et al., 2019; Akosa, 2017). The GM is defined as the square root of the product of the sensitivity (true positive rate) and specificity (false positive rate) and is defined as follows:

(5) $$GM = \sqrt {sensitivity*specificity} = \sqrt {{{TP} \over {TP + FN}}*{{TN} \over {TN + FP}}} ,$$

where TP and TN represent the numbers of true positives and true negatives, respectively. Similarly, FP denotes the number of false positives, and FN represents the number of false negatives. Note that the value of the GM score is reduced to zero if the sensitivity score of one of the classes under observation is equal to zero.

The ROC curve was determined by plotting the TP rate against the FP rate at different threshold levels. The ROC AUC score was then computed as the area under the ROC curve, taking values between 0 and 1. The ROC AUC measure also takes the prediction accuracies achieved for both classes into account, thereby also preventing the result from being biased toward the majority class.

### Results

An overview of the best achieved GM scores in the experiments performed on agriculture and construction data are depicted in Table 4. The most promising results were achieved by the STE, MTE and SDA models, where the prediction performance in terms of the GM score reached 85.6%. For agriculture, superior results were produced by the MTE models using data from years ${R_{eval - 3}}$ containing 60 features. Here, the best GM scores ranged from 76.4% to 81.1%. Competitive results were observed for the MTE models using ${R_{eval - 1}}$ (20 features) and ${R_{eval - 2}}$ data (40 features). Slightly decreased prediction performances were achieved by the SDA and STE models. The application of the SVM, DT and XGB methods resulted in poor model performance. This was probably caused by the models’ equal data distribution assumption that often results in a bias toward the majority class samples.

**Table 4:** The best GM scores (in %) achieved on data from the agriculture and construction industries (± stands for standard deviation).

		Agriculture				Construction			
		2013	2014	2015	2016	2013	2014	2015	2016
${R_{eval\_1}}$	STE	58.8 ± 11	65.2 ± 2	75.6 ± 2	75.3 ± 4	69.8 ± 4	67.5 ± 6	75.3 ± 4	79.5 ± 3
	MTE	77.3 ± 7	74.4 ± 4	74.8 ± 4	**77.3** ± **4**	**72.7** ± **3**	70.5 ± 6	81.2 ± 3	**85.6** ± **3**
	SDA	66.6 ± 5	66.5 ± 2	75.3 ± 2	74.0 ± 4	69.6 ± 4	67.7 ± 7	76.5 ± 4	77.4 ± 2
	DT	5.0 ± 22	15.0 ± 36	7.1 ± 21	10.6 ± 25	34.9 ± 24	24.5 ± 20	24.5 ± 28	23.0 ± 28
	SVM	0.0 ± 0	9.9 ± 30	17.6 ± 31	14.1 ± 28	2.2 ± 10	2.0 ± 9	22.0 ± 28	8.7 ± 21
	XGB	0.0 ± 0	60.0 ± 50	21.2 ± 33	7.1 ± 22	48.2 ± 19	49.7 ± 18	49.0 ± 19	39.9 ± 31
${R_{eval\_2}}$	STE	64.5 ± 10	64.4 ± 3	74.1 ± 5	67.8 ± 4	70.8 ± 2	61.6 ± 8	**82.9** ± **2**	73.4 ± 2
	MTE	71.0 ± 7	70.9 ± 4	76.0 ± 6	71.6 ± 4	71.4 ± 4	**71.0** ± **5**	80.9 ± 3	81.3 ± 6
	SDA	67.1 ± 9	66.6 ± 2	76.5 ± 4	70.3 ± 3	71.4 ± 2	65.9 ± 7	78.4 ± 2	74.0 ± 3
	DT	5.0 ± 22	10.0 ± 30	10.6 ± 25	14.1 ± 28	37.7 ± 23	38.2 ± 28	47.9 ± 26	24.3 ± 30
	SVM	4.9 ± 21	14.8 ± 35	21.1 ± 32	28.2 ± 28	18.7 ± 23	10.2 ± 18	31.9 ± 27	17.3 ± 26
	XGB	5.0 ± 22	40.0 ± 50	24.7 ± 35	10.6 ± 26	54.1 ± 13	51.4 ± 21	53.2 ± 21	49.8 ± 27
${R_{eval\_3}}$	STE	75.8 ± 4	68.1 ± 2	71.9 ± 3	70.1 ± 3	69.1 ± 2	65.4 ± 5	80.6 ± 3	70.7 ± 3
	MTE	**81.1 ± **5****	**76.4** ± **2**	**78.1** ± **9**	76.2 ± 5	67.7 ± 3	64.7 ± 7	82.8 ± 3	79.8 ± 3
	SDA	73.8 ± 5	66.2 ± 5	72.1 ± 3	71.4 ± 3	70.8 ± 1	65.6 ± 5	80.2 ± 3	70.1 ± 2
	DT	9.9 ± 30	25.0 ± 43	10.6 ± 25	7.1 ± 21	42.6 ± 24	37.8 ± 23	35.2 ± 27	30.0 ± 30
	SVM	5.0 ± 22	34.7 ± 47	21.0 ± 32	31.5 ± 35	35.5 ± 25	26.0 ± 22	35.7 ± 31	23.0 ± 28
	XGB	10.0 ± 31	40.0 ± 50	21.2 ± 33	10.6 ± 26	53.6 ± 18	41.8 ± 19	54.1 ± 9	36.2 ± 35

**Note:**

The best results are highlighted in bold in each column.

As in the agriculture scenario, the application of the MTE to construction also led to the best results in terms of the GM score in the majority of cases. The highest prediction performance was 85.6% for the model derived from ${R_{eval - 1}}$ data for ${R_{eval}}$ = 2016. The application of the STE for ${R_{eval}}$ = 2015 slightly outperformed the MTE approach while ${R_{eval - 2}}$ data were used. The results obtained for ${R_{eval}}$ = 2013 and ${R_{eval}}$ = 2016 yielded the best prediction performance when ${R_{eval - 1}}$ data were utilized. Our results suggest that the data from the period one year prior to bankruptcy are the most indicative data of upcoming financial problems. On the other hand, the model using 40 features ( ${R_{eval - 2}}$) for ${R_{eval}}$ = 2014 and ${R_{eval}}$ = 2015 slightly outperformed the models derived from ${R_{eval - 1}}$ data. The rest of the utilized algorithms (the SVM, the DT and XGB) achieved significantly lower overall performance with higher standard deviations; thus, these algorithms were deemed to be inferior in bankruptcy prediction tasks.

The results of experiments conducted on the manufacturing industry are depicted in Table 5. The best results were obtained by the MTE model with a GM score of up to 80.6% on the ${R_{eval - 1}}$ data. The MTE model outperformed the other models in all evaluation years except ${R_{eval}}$ = 2014, where the STE model achieved a more accurate prediction. These results proved our assumption that an ensemble of shallow autoencoders is able to identify bankrupt companies. The models based on the SVM, the DT and XGB performed poorly, as in the agriculture and construction cases, with GM scores not exceeding 35%.

**Table 5:** The best GM scores (in %) achieved on data from the manufacturing and retail industries (± stands for standard deviation).

		Manufacturing				Retail			
		2013	2014	2015	2016	2013	2014	2015	2016
${R_{eval\_1}}$	STE	71.1 ± 4	**73.6** ± **3**	72.9 ± 3	76.3 ± 5	63.7 ± 6	62.0 ± 4	84.5 ± 2	74.4 ± 5
	MTE	71.1 ± 3	73.3 ± 4	74.9 ± 3	**80.6** ± **4**	62.8 ± 8	65.8 ± 3	**93.7** ± **3**	**84.5** ± **5**
	SDA	69.8 ± 4	69.9 ± 3	69.3 ± 3	76.8 ± 5	54.0 ± 8	63.2 ± 4	88.4 ± 4	80.7 ± 5
	DT	12.2 ± 19	17.8 ± 23	35.4 ± 31	11.5 ± 23	21.2 ± 32	15.6 ± 32	5.0 ± 22	25.0 ± 43
	SVM	0.0 ± 0	4.1 ± 12	8.9 ± 18	8.7 ± 21	3.5 ± 15	3.5 ± 15	0.0 ± 0	30.0 ± 46
	XGB	28.2 ± 22	36.5 ± 23	47.9 ± 21	12.7 ± 27	24.7 ± 35	38.9 ± 37	30.0 ± 47	45.0 ± 50
${R_{eval\_2}}$	STE	72.0 ± 3	68.6 ± 2	75.7 ± 2	72.2 ± 2	69.6 ± 3	66.6 ± 4	87.6 ± 2	78.5 ± 5
	MTE	**73.0** ± **4**	72.6 ± 3	**77.9** ± **3**	79.1 ± 4	**71.3** ± **4**	70.5 ± 3	90.4 ± 5	79.8 ± 5
	SDA	71.0 ± 3	69.4 ± 3	69.7 ± 3	73.7 ± 2	66.3 ± 4	67.8 ± 4	89.0 ± 3	80.3 ± 5
	DT	6.1 ± 15	16.3 ± 20	28.3 ± 26	5.8 ± 17	14.1 ± 28	10.6 ± 25	15.0 ± 36	20 ± 43
	SVM	8.1 ± 16	10.2 ± 18	15.6 ± 21	5.8 ± 17	7.1 ± 21	17.7 ± 31	0.0 ± 0	39.9 ± 49
	XGB	29.9 ± 23	40.4 ± 20	51.0 ± 25	17.3 ± 27	38.8 ± 36	35.3 ± 36	30.0 ± 47	10.0 ± 31
${R_{eval\_3}}$	STE	69.5 ± 2	69.8 ± 2	72.1 ± 2	70.0 ± 2	66.3 ± 4	64.7 ± 4	86.9 ± 4	77.9 ± 5
	MTE	72.8 ± 5	72.6 ± 3	76.2 ± 4	76.9 ± 7	69.8 ± 3	**71.9** ± **3**	86.7 ± 6	79.3 ± 3
	SDA	69.5 ± 2	66.7 ± 2	69.9 ± 1	72.6 ± 2	65.5 ± 3	66.2 ± 3	85.9 ± 2	76.3 ± 4
	DT	10.2 ± 18	12.2 ± 19	29.6 ± 25	2.9 ± 13	7.0 ± 21	3.5 ± 15	5.0 ± 22	30.0 ± 46
	SVM	13.1 ± 20	8.2 ± 16	24.6 ± 22	14.4 ± 25	14.1 ± 28	14.1 ± 28	5.0 ± 22	55.0 ± 50
	XGB	29.0 ± 23	33.4 ± 18	49.5 ± 16	27.1 ± 21	24.7 ± 35	35.3 ± 36	40.0 ± 50	5.0 ± 22

**Note:**

The best results are highlighted in bold in each column.

**Figure 5:**
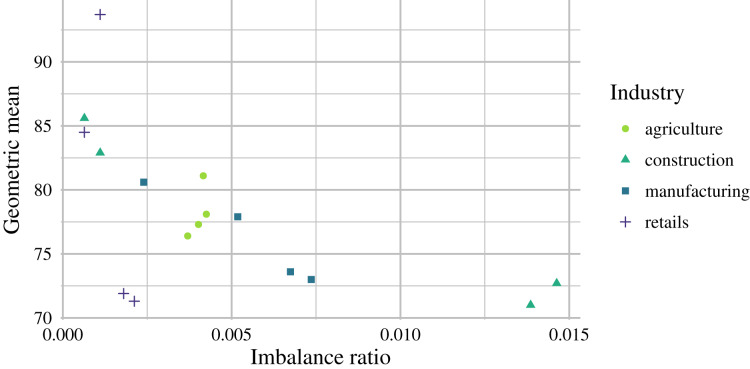
Relationship between sample imbalance (in %) and model performance (GM) for the autoencoder ensembles for the analyzed industries.

The last set of experiments was conducted on retail, and the results are depicted in the second part of Table 5. In this case, the MTE model yielded superior results across all utilized evaluation years. The highest achieved prediction performance was obtained for ${R_{eval}}$ = 2015 (GM = 93.7%), followed by that obtained with ${R_{eval}}$ = 2016 (GM = 84.5%), both of which were derived from ${R_{eval - 1}}$ data. For retail, the prediction performance rapidly improved when data after ${R_{eval}} = 2014$ were utilized. Interestingly, better results were observed for evaluation years with higher levels of class imbalance (Fig. 5). Furthermore, the GM scores of the models derived from the STE, MTE and SDA architectures seldom dropped below 80%.

From the practical point of view, only marginal prediction performance improvements were obtained when ${R_{eval - 2}}$ or ${R_{eval - 3}}$ data were used compared to the results obtained with ${R_{eval - 1}}$ data. In real-world applications, it might be difficult or not economically viable to use a longer timeframe than ${R_{eval - 1}}$ for such a small prediction improvement considering data availability and computational difficulty. On average, the best GM scores were as follows: 78.23% for agriculture, 78.05% for construction, 76.28% for manufacturing, and 80.35% for retail. No significant prediction performance differences were observed between the analyzed industries; however, retail scored best. Prediction performance differences over the years may have been caused by macroeconomic development or other factors, which are not reflected in financial ratios. By comparing the prediction performance and levels of data imbalance, we can see a negative correlation in Fig. 5, which indicates that the proposed models are suitable for highly imbalanced data.

For comparison, Tables 6 and 7 depict the best AUC scores produced for all industries. In general, the AUC scores are higher than the GM scores, which can be explained by their metric definitions. The results show that the GM is a more suitable metric for highly class-imbalanced data because it takes misclassification into consideration for both classes. The AUC scores are presented for better comparability since the majority of studies use this metric.

**Table 6:** The best AUC scores (in %) achieved on data from the agriculture and construction industries (± stands for standard deviation).

		Agriculture				Construction			
		2013	2014	2015	2016	2013	2014	2015	2016
${R_{eval\_1}}$	STE	63.4 ± 7	65.3 ± 2	75.9 ± 2	76.1 ± 3	70.0 ± 4	69.1 ± 4	77.4 ± 3	80.2 ± 2
	MTE	78.2 ± 6	75.2 ± 4	75.1 ± 3	**78.6** ± **4**	**73.2** ± **3**	**72.7** ± **4**	81.7 ± 3	**85.9** ± **2**
	SDA	68.3 ± 4	66.5 ± 2	75.6 ± 1	74.4 ± 4	69.9 ± 4	69.1 ± 5	78.4 ± 3	77.8 ± 2
	DT	52.2 ± 11	57.3 ± 18	52.3 ± 8	53.6 ± 9	58.6 ± 7	60.5 ± 7	56.6 ± 8	56.5 ± 8
	SVM	50.0 ± 0	54.7 ± 15	56.1 ± 11	54.8 ± 10	50.3 ± 2	50.3 ± 2	56.1 ± 8	52.5 ± 6
	XGB	63.2 ± 7	**80.0** ± **25**	57.4 ± 12	52. ± 8	60.2 ± 10	63.2 ± 7	63.6 ± 7	62.5 ± 11
${R_{eval\_2}}$	STE	66.5 ± 7	64.6 ± 3	75.0 ± 4	70.0 ± 0	71.3 ± 2	64.9 ± 6	**83.2** ± **2**	73.8 ± 2
	MTE	72.9 ± 6	71.1 ± 4	76.6 ± 5	72.2 ± 4	72.4 ± 4	72.2 ± 4	81.2 ± 3	82.3 ± 5
	SDA	68.8 ± 7	66.7 ± 2	77.2 ± 3	71.4 ± 3	71.5 ± 2	67.3 ± 5	79.2 ± 2	74.4 ± 3
	DT	52.2 ± 11	54.7 ± 15	53.6 ± 9	54.8 ± 10	59.4 ± 8	60.7 ± 10	64.7 ± 10	57.4 ± 10
	SVM	51.7 ± 11	56.8 ± 18	57.1 ± 11	59.4 ± 12	53.8 ± 6	51.7 ± 4	58.4 ± 8	54.7 ± 8
	XGB	65.4 ± 8	70.0 ± 25	58.6 ± 12	53.7 ± 9	60.8 ± 10	65.4 ± 8	66.1 ± 8	65.8 ± 10
${R_{eval\_3}}$	STE	76.4 ± 4	68.1 ± 2	72.1 ± 3	70.6 ± 3	69.2 ± 2	66.3 ± 4	81.2 ± 2	71.0 ± 2
	MTE	**81.6** ± **4**	77.2 ± **2**	**79.1** ± **7**	76.7 ± 5	68.8 ± 2	68.3 ± 5	83.1 ± 3	80.2 ± 3
	SDA	73.9 ± 5	66.7 ± 4	72.3 ± 3	71.7 ± 3	70.9 ± 1	66.6 ± 4	80.9 ± 3	71.3 ± 2
	DT	54.6 ± 15	62.3 ± 22	53.6 ± 9	52.3 ± 8	61.6 ± 9	59.5 ± 7	59.6 ± 9	58.9 ± 10
	SVM	51.5 ± 11	66.6 ± 24	56.5 ± 12	60.4 ± 12	58.7 ± 8	55.2 ± 5	60.5 ± 11	56.1 ± 8
	XGB	65.9 ± 9	70.0 ± 25	57.5 ± 12	53.7 ± 9	59.4 ± 10	65.9 ± 9	64.9 ± 5	62.5 ± 14

**Note:**

The best results are highlighted in bold in each column.

**Table 7:** The best AUC scores (in %) achieved on data from the manufacturing and retail industries (± stands for standard deviation).

		Manufacturing				Retail			
		2013	2014	2015	2016	2013	2014	2015	2016
${R_{eval\_1}}$	STE	71.4 ± 4	73.8 ± 3	73.5 ± 2	78.3 ± 3	65.4 ± 4	63.9 ± 4	85.6 ± 2	75.0 ± 6
	MTE	71.4 ± 3	**74.0 ± 3**	75.2 ± 3	**81.5 ± 3**	67.2 ± 5	69.8 ± 3	**93.8 ± 2**	**84.8 ± 5**
	SDA	70.0 ± 4	70.8 ± 3	69.9 ± 2	79.0 ± 3	60.6 ± 5	66.4 ± 3	88.7 ± 4	80.9 ± 6
	DT	52.2 ± 4	54.1 ± 6	60.9 ± 11	53.3 ± 7	57.4 ± 11	56.2 ± 13	52.5 ± 11	62.5 ± 22
	SVM	50.0 ± 0	50.8 ± 2	52.0 ± 4	52.4 ± 6	51.2 ± 5	51.2 ± 5	0.5 ± 0	65.0 ± 23
	XGB	56.2 ± 5	59.1 ± 7	63.4 ± 9	54.1 ± 9	58.7 ± 12	63.7 ± 13	65.0 ± 24	72.5 ± 26
${R_{eval\_2}}$	STE	72.5 ± 3	68.8 ± 2	76.2 ± 2	73.5 ± 2	70.7 ± 3	68.4 ± 3	88.2 ± 2	78.8 ± 5
	MTE	73.5 ± 4	73.2 ± 3	**78.2 ± 3**	79.5 ± 4	**72.4 ± 3**	72.1 ± 3	90.7 ± 5	80.2 ± 5
	SDA	71.4 ± 3	69.7 ± 3	69.9 ± 3	75.4 ± 2	68.7 ± 3	69.7 ± 3	89.4 ± 3	80.6 ± 3
	DT	51.0 ± 3	53.2 ± 4	57.4 ± 8	51.6 ± 5	54.9 ± 10	53.7 ± 9	57.4 ± 8	60.0 ± 20
	SVM	51.5 ± 3	52.0 ± 4	53.4 ± 5	51.6 ± 5	52.4 ± 7	56.1 ± 11	50.0 ± 0	69.9 ± 24
	XGB	57.0 ± 6	60.0 ± 6	66.0 ± 10	55.0 ± 8	63.8 ± 13	62.5 ± 13	65.0 ± 24	55.0 ± 15
${R_{eval\_3}}$	STE	71.4 ± 2	70.2 ± 2	73.9 ± 2	72.5 ± 3	67.1 ± 3	66.5 ± 3	87.6 ± 4	78.3 ± 5
	MTE	**74.0** ± **3**	73.7 ± 3	76.7 ± 4	78.8 ± 5	71.3 ± 2	**73.1** ± **2**	87.0 ± 6	78.3 ± 5
	SDA	70.0 ± 2	67.0 ± 2	70.5 ± 2	74.2 ± 3	66.4 ± 3	67.5 ± 2	86.9 ± 2	76.8 ± 5
	DT	51.9 ± 4	52.4 ± 4	57.4 ± 7	50.8 ± 4	52.4 ± 7	51.2 ± 5	52.4 ± 11	65.0 ± 23
	SVM	52.7 ± 5	51.5 ± 3	55.3 ± 5	54.0 ± 7	54.8 ± 10	54.9 ± 10	52.3 ± 11	77.5 ± 25
	XGB	56.6 ± 6	57.0 ± 4	63.5 ± 8	58.3 ± 10	58.8 ± 12	62.5 ± 13	70.0 ± 25	52.5 ± 11

**Note:**

The best results are highlighted in bold in each column.

## Conclusions

Currently, data distribution skewness is a crucial issue in many machine learning domains, and bankruptcy prediction is no exception. In this article, we present an approach based on an ensemble of autoencoders that can cope with the highly imbalanced nature of data. We designed and comparatively analyzed two approaches based on ensembles of shallow autoencoders, namely, an STE and an MTE. Furthermore, the SDA was applied for comparison purposes.

For numerical experiments, we used data that were composed of thousands of financial ratios of small and medium-sized companies operating in the Slovak Republic during the years 2010–2016. We noticed that the MTE performed much better than the STE and SDA. For the majority of the datasets, the MTE approach yielded the highest GM scores. The highest prediction score obtained by the MTE was 93%. However, the achieved results varied across the considered evaluation years and industries. As expected, the application of the reference machine learning algorithms (the SVM, the DT and XGB) resulted in poor model performance across all utilized datasets. The ineffectiveness of the selected reference algorithms was probably caused by the assumption of a balanced sample distribution, which often results in a bias toward the majority class in severely imbalanced scenarios.

The experiments proved that the proposed MTE approach is able to handle highly imbalanced data. Even in this challenging scenario, the approach identified bankrupt companies. Moreover, we utilized only well-known financial attributes that could be obtained from companies’ annual reports. Finally, even though we built an ensemble of NNs, the base classifiers were not deep, so the MTE approach is not computationally expensive.

In our study, we used the financial ratios of the SMEs in four different industries: agriculture, construction, manufacturing, and retail. Some specifics should be noted regarding the financial reporting of SMEs, for example, the yearly frequency of reporting (which is longer than that of companies listed on the stock market) and the level of information that is reported by SMEs compared to that of large companies. Smaller companies are usually not obligated to comply with international accounting standards (*i.e*., IFRS), and their annual reports do not have to be confirmed by independent auditors. The ratio between bankrupt and nonbankrupt companies is small; therefore, the available data may not provide a recognizable pattern, not even for machine learning methods. There are some events (such as pandemics, financial crises, energy crises, *etc*.) that occur irregularly but have extensive global influences on different aspects of the economy. Such events may have different impacts on individual industries and may be manifested at different times for each sector.

Our findings are of interest to financial institutions, rating agencies, and business partners who mainly depend on publicly available information from annual reports. The proposed methods can be implemented in business intelligence systems for the automatic evaluation of a company’s bankruptcy status based on periodically published yearly data. The main disadvantage is that although methods based on the financial ratios of companies are considered robust in general, they may neglect some macroeconomic developments (*i.e*., inflation, unemployment, or even recession). For future work, we propose to evaluate the developed model in different countries and over longer time frames.

Even though autoencoder-based models performed better than reference algorithms such as the SVM, the DT and XGB, some caveats need to be mentioned. First, the training time of the proposed models is much more resource-sensitive than those of the reference algorithms because it contains many internal parameters that need to be set using the backpropagation algorithm. Second, the architectures of the autoencoder-based models depend on the given hyperparameters. Choosing the optimal combination of hyperparameters and evaluating model performance is time-consuming, especially in the case of the MTE model, which is tuned by a GA.

## Supplemental Information

10.7717/peerj-cs.1257/supp-1 Supplemental Information 1 Raw bankruptcy data.

## Additional Information and Declarations

### Data Availability

The following information was supplied regarding data availability: Data is available in the Supplemental Files, GitHub, and Mendeley: https://github.com/kanasz/Bankruptcy_Prediction. Drotár, Peter; Gnip, Peter; Zoričak, Martin; Gazda, Vladimir (2019), “Dataset of financial ratios of Slovak companies”, Mendeley Data, V2, DOI 10.17632/j89csb932y.2. The script for running experiments (Ensemble_Main.py) is also available at GitHub: https://github.com/kanasz/Bankruptcy_Prediction/tree/main/Classifiers/Ensemble_Autoencoder.
